# Predation threat affects isotope ratios of planktonic consumers

**DOI:** 10.1007/s00442-025-05844-8

**Published:** 2025-12-08

**Authors:** Tomasz Brzeziński, M. Bojanowski, M. Radzikowska

**Affiliations:** 1 Department of Hydrobiology, Faculty of Biology, University of Warsaw, Building CENT3 Room 5.04, Żwirki I Wigury 101, 02-089 Warsaw, Poland; 2 Institute of Geological Sciences, Polish Academy of Sciences, Twarda 51/55, 00-818 Warsaw, Poland

**Keywords:** Stable isotope analysis, Trophic isotope enrichment, Zooplankton, Stress, Ecological indicators

## Abstract

The analysis of stable isotopes of carbon and nitrogen is widely used in ecological investigations. However, sources of variation in isotope ratios of animals are far from being identified, which may bias the interpretation of the results. It has been suggested that stress is a factor that affects isotope ratios and trophic enrichment in animal bodies. Here, we tested the hypothesis that stress due to exposure to a predation threat affects metabolic processes that ultimately result in altered isotope composition of threatened prey. We found that both the nitrogen isotope composition (δ^15^N) and trophic enrichment in ^15^N isotope (Δ^15^N) were affected by threat induced by planktivorous fish in two species of the keystone freshwater pelagic herbivore *Daphnia*: *D. magna* and *D. pulex*. The two species differed from each other with regard to isotope ratios. Despite this, with increasing perceived risk of predation, the δ^15^N decreased in both species and, as a consequence, individuals of the two species were depleted in ^15^N with respect to their conspecifics not exposed to predation. We have not found evidence that the carbon isotope ratio (δ^13^C) was affected by the predation threat. The eco-physiological responses underlying the induction of antipredatory defences may be responsible for the observed pattern. The effect of predation on the nitrogen isotope ratio (δ^15^N and Δ^15^N) of consumers should be accounted for when using the analysis of stable isotopes in ecological investigations. Moreover, it could be applied to the fossil record to identify the introduction or removal of planktivorous predators in the paleoenvironment.

**Supplementary Information:**

The online version contains supplementary material available at 10.1007/s00442-025-05844-8.

## Introduction

Stable isotope analysis (SIA) is a powerful tool used to gain insights into various ecological processes, including food web dynamics, trophic interactions, nutrient cycling, migration patterns, and environmental change (Grey 2006; Boecklen et al. 2011; Shipley and Matich 2020; Hobson 2023). The number of studies using SIA for these purposes published in peer-reviewed journals has increased exponentially. This is due to several advantages of this method over traditional ones (Boecklen et al. 2011; Shipley and Matich 2020; Hobson 2023), for example, assessment of trophic linkages in pelagic systems using traditional methods is not easy (limited possibility of direct observations, gut content frequently liquefied or too digested to permit identification); moreover, isotope ratios of consumer tissues provide a time-integrated reflection of the diet, a significant improvement compared to a snapshot of the last meal that can be obtained from gut content analysis (Grey 2006). Yet, understanding the reasons influencing stable isotope ratio and fractionation of isotopes on the resource-consumer interface is far from complete, even in the field of identification of trophic relations, where SIA is used most frequently. According to the central paradigm of ecological applications of the SIA, “*you are what you eat plus a few per mil*”, which denotes that isotope composition of carbon (δ^13^C) and nitrogen (δ^15^N) in an organism’s tissues reflect those of its dietary sources and enrichment of the heavier isotope in consumer’s tissues due to metabolic processes (Gannes et al. 1998). Despite that, relatively little is known about the sources of variation in the stable isotope ratios of animals observed in natural populations. This limits the interpretation of results acquired using SIA (Grey 2006; Auerswald et al. 2010; Boecklen et al. 2011).

Although it is assumed that the isotope ratios of consumers depend on the isotope ratios of food (bottom-up effect), theoretical considerations and empirical evidence point to the fact that there are multiple sources, not only baseline food isotopic composition, that contribute to the observed variance of isotope ratios in consumers (Boecklen et al. 2011; Shipley and Matich 2020). Changes in isotope ratios ultimately depend on physiological processes in consumer bodies, which in turn are strongly affected by several ecological factors. Ambient temperature affects the trophic isotope enrichment of consumers—with increasing temperature, the animals tend to be more enriched in heavier isotopes, which may stem from increasing metabolic rate (Gannes et al. 1998). It has been shown that physiological stress, driven by exposure to unfavorable environmental conditions, also influences isotope ratios. In starving animals, the tissues become progressively enriched in ^15^N and ^13^C due to the preferential excretion of light isotopes, ^14^N and ^12^C respectively, during urea formation after deamination of amino-acids, and the mobilization of stored lipids, which contain relatively more ^12^C (Hobson et al. 1993; Gannes et al. 1998). Organisms exposed to environmental pollution express altered isotope ratios. Fish living in wastewater-polluted habitats showed ^15^N enrichment compared to fish from non-polluted habitats (Schlacher et al. 2005). Cladocerans exposed to the toxic organoarsenic also showed higher ^15^N enrichment than their conspecifics raised in a toxin-free environment (Brzeziński et al. 2020). One may expect that other ecological factors that affect the physiology of an individual may ultimately influence its isotope composition of carbon and nitrogen. If so, estimates of the diet composition or trophic position of animals based on isotope composition should account for this.

Predation is one of the most important organizing factors in ecology. It is ubiquitous in aquatic environments, although there are habitats free of predators (Gliwicz 2003), and its strength varies in space and time (Sommer et al. 2012). Indirect, non-lethal effects of predation on prey are as important as direct (consumptive) ones. The threat of predation can drive changes in prey physiology, development, morphology, and behavior (Gliwicz 2003; Clinchy et al. 2013; Sheriff and Thaler 2014). The indirect effects of predation are particularly well recognized in aquatic environments, where infochemicals released into the water (kairomones and alarmones) can be detected by prey, triggering the expression of defensive mechanisms (Von Elert and Loose 1996; Pijanowska 1997). In planktonic cladocerans, which are subjected to predation by planktivorous fish, these infochemicals induce a wide array of adaptive responses that include reduction of body size, development of morphological defensive structures, and modification of life histories (e.g., earlier maturation and production of smaller but more abundant offspring) (Gliwicz 2003). These effects elicited in cladocerans by exposure to predator threat are associated with profound alterations in biochemical architecture and physiological processes. These include altered gene expression, changes in the cytoskeleton (Pijanowska and Kloc 2004; Colbourne et al. 2011), metabolic rate (Robinson et al. 2018), altered efficiency of energy acquisition, and a switch in the allocation of energy from investment into somatic growth to boosting reproduction (Gliwicz 2003; Rinke et al. 2008). So far, it is not clear if all the mentioned effects may affect the isotope ratios of animals. It is known, however, that different types of tissues are characterized by different isotope ratios (isotope routing, Gannes et al. 1998). Since predation threat affects energy allocation between different types of tissues and body structures, it is not unlikely that this will ultimately result in changes in the isotope composition of the threatened individual compared to the not-threatened one. However, the effects of the threat of predation on the stable C and N isotope composition of the prey have not been investigated.

Planktivorous fish are visual predators; they select for larger individuals (species) of zooplankton (e.g.Brooks 1968; Gliwicz 2003). Small-bodied zooplankton are harder to detect by fish; at the same time, large-bodied individuals are more profitable food items (see e.g., Gliwicz 2003 for review). Consequently, the risk of being hunted by fish is higher in large-bodied individuals than in small-bodied ones (e.g., Leibold and Tessier 1991), and accordingly to the perceived risk of predation, individuals adjust the development of antipredatory defensive mechanisms (Pijanowska 1997; Hansson and Hylander 2009). We suspected that, due to the difference in perceived risk of predation, the effects of predation threat on isotopic ratios in zooplankton prey may depend on body size—in small-bodied individuals, which are less threatened, isotopic ratios may be less affected.

This study aims to test if the threat of predation might affect the isotope composition of carbon and nitrogen of the prey. We hypothesize that (1) isotope ratios of consumers raised in the presence of cues of predation differ from those unexposed to cues of predators, (2) the effect of predation threat on isotope ratios of the prey depends on the intensity of the threat (density of the predator), and (3) the effect of the predation threat on the isotopic ratio will be more pronounced in large-bodied species (which are more prone to predation) than in small-bodied ones. Testing these hypotheses may verify if the isotope composition of organisms could be used as an ecological indicator of stress caused by the threat of predation. This may also provide important data that allows reducing bias in the estimation of trophic discrimination factors used to determine trophic position.

## Materials and methods

### Organisms

We determined life history parameters (somatic growth rate, fecundity, and size at first reproduction) and isotope composition (δ^13^C and δ^15^N) of one clone of *D. magna* Straus, 1820, and one clone of *D. pulex* Leydig, 1860, cultivated in the absence or in the presence of chemical cues (kairomones and alarm substances) released by planktivorous fish (roach, *Rutilus rutilus* (L.)). The first clone (further referred to as DMB) originated from Grosser Binnensee (NE Germany), a brackish lake, and the second one (further referred to as DPM1) originated from a pond near Lake Mikołajskie (NE Poland). Both water bodies are, at least temporarily, occupied by fish. Representatives of two species of *Daphnia* were selected to account for the fact that species within the genus differ from each other with regard to body size, and these differences affect their performance (i.e., filtration rate, efficiency of energy assimilation, susceptibility to predation) (Gliwicz 2003; Maszczyk and Brzeziński 2018). The size (length) of individuals of the DMB clone at first reproduction is approximately 1/3 larger than those of the DPM1 clone. The size of the smaller species allowed for obtaining replicable results of the analysis of stable isotopes with 2 individuals per sample (~ 100 μg dry weight).

### Maintenance of daphnids

The clones were maintained at the facilities of the Department of Hydrobiology, University of Warsaw. The animals were cultivated in a mixture of conditioned lake water and distilled water (1:1). Lake water originated from Lake Szczęśliwickie (a small, stratified lake located in Warsaw, Poland), and was stored for more than a month in a 10 m^3^ tank in the facilities of the Department of Hydrobiology. The water in the tank was kept in darkness and aerated. The water used in the experiment was free of organic pollutants; the concentration of heavy metals was very low—much below the toxicity thresholds for daphnids, and the nutrient level was high, typical for an eutrophic lake. We found no differences with regard to the analyzed chemical parameters of water between fish and fish-free treatments (see Supplementary data for detailed chemical characteristics of water used in the experiment).

### Experimental set-up

#### Preparation of experimental media

**Fig. 1:**
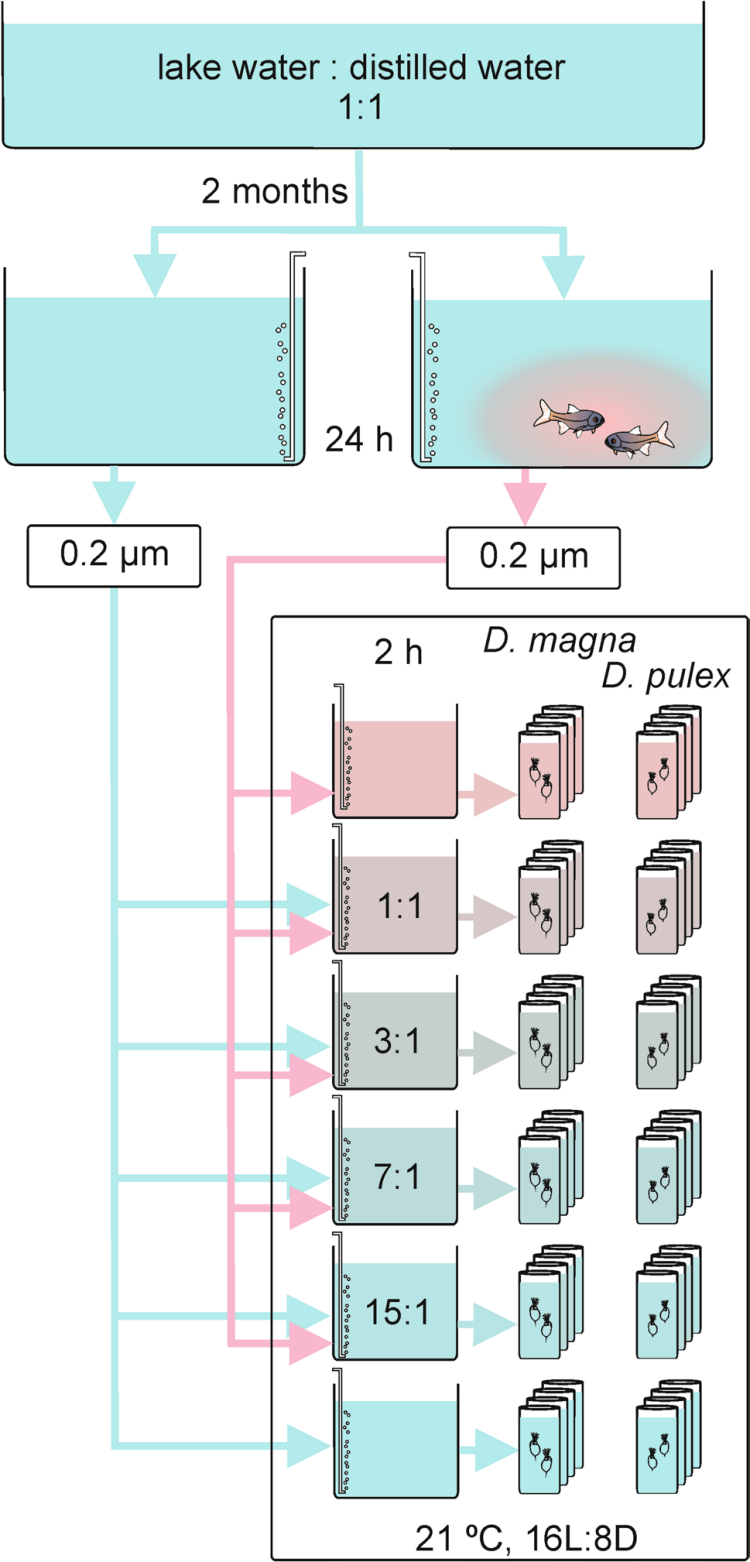
Schematic representation of the experimental setup

Media for cultivation of animals and for the experiment were prepared using the same batch of lake water. The schematic representation of media preparation is shown in Fig. 1.

Five concentrations (dilutions) of fish-conditioned water were tested, corresponding to a density of 0.5 fish^.^L^−1^, 0.25 fish^.^L^−1^, 0.125 fish^.^L^−1^, 0.0625 fish^.^L^−1^, and 0.0312 fish^.^L^−1^. A sixth treatment (control) was prepared free of fish infochemicals.

Control medium constituted a mixture of lake water and distilled water (proportion 1:1); it was aerated without the presence of fish for 24 h before the experiment. Simultaneously, fish-conditioned water was prepared by exposing two individuals of roach (*Rutilus rutilus* (L.), 6–7 cm body length) in 4 L of the same batch of the mixture of lake water and distilled water for 24 h. Roach is frequently used as a source of kairomones for studying reactions of *Daphnia* to fish predation (e.g. Ślusarczyk 1999; Burks et al. 2000), in lakes of Masurian Lakeland, it is one of the principal planktivores (e.g. Gliwicz et al. 2000). The medium in the fish aquarium was aerated and renewed daily. Living daphnids (mixture of both species, in approximately equal proportions) were used to feed fish during the experiment—thus the fish-conditioned medium contained both fish kairomones and daphnids’ alarm substances. Fish were maintained in the same lab and in the same conditions as daphnids but in a separate water bath (temperature 21 ± 0.1 °C, summer photoperiod). These conditions corresponded to the summer temperature of the epilimnion of Lake Roś (Masurian Lakeland, NE Poland), from which roaches were collected. Control and fish-conditioned water were filtered through 0.2 μm pore size membrane filters to remove particulate organic matter, including bacteria. Control water was used to dilute fish-conditioned water to obtain concentrations of kairomones and alarmones corresponding to the chosen fish densities (no dilution of fish-conditioned water, 1:1, 1:3, 1:7, and 1:15 dilution, respectively). Prepared media were aerated for 2 h in a water bath (21 ± 0.1 °C) before adding food suspensions and transferring daphnids.

#### Preparation of food suspensions for daphnids

The food source was *Chlamydomonas klinobasis* (strain 56, Limnological Institute, University of Cologne) from a stationary phase, chemostat culture grown in WC medium (Guillard 1975) at a constant temperature of 19 ± 0.5 °C. The carbon content of the algal suspension was determined by reference to photometric light extinction at 800 nm using Lambda EZ201 spectrophotometer (Perkin-Elmer, USA), along with carbon-extinction equations determined previously. The carbon content was measured by the dry combustion method using a Flash 2000 Elemental Analyzer (Thermo Fisher Scientific Inc., USA).

#### Running the experiment

Synchronized cohorts of 12 h-old neonates were used to start the growth experiments. Initial weight was measured in two subsamples of 9–10 individuals. The experimental animals originated from the third broods of mothers reared under non-limiting concentrations of *Ch. klinobasis* for three generations. The experiments were conducted in beakers filled with 0.4 L of medium, with a food level of 1.5 mg C L^−1^. Two animals were kept in each beaker, each treatment consisted of four replicates (beakers) per clone. Each clone was tested independently. Every day, fresh medium for each treatment was prepared, and animals were transferred to new beakers with fresh medium. Beakers and pipettes were cleaned and autoclaved (132 °C) to minimize bacterial growth. Beakers with animals were kept in a water bath at a constant temperature of 21 ± 0.1 °C. The temperature was maintained constant using Zefir (Adarex, Poland) submersible thermostats. The light conditions simulated the summer photoperiod (16:8 D:N). Light intensity, measured using a LI-COR 189 quantum sensor (LI-COR Biosciences^®^, USA), was 0.32 ± 0.04 µmol × m^−2^ × s^−1^.

The animals were kept in beakers until they released the first clutch into the brood chamber. Eggs in the brood chambers were counted under a dissecting microscope, the length of the animals (size at first reproduction, SFR) was measured from the base of the tail spine to the upper margin of the compound eye to the nearest 0.01 mm. The animals were then transferred to preweighed tin boats (one boat per beaker), dried at 60 °C for 48 h, cooled in a desiccator, and weighed on an Orion Cahn C-35 electrobalance (Thermo Electron Corporation, USA) to the nearest 0.1 μg. Initial weight was measured in two subsamples of 9–10 individuals. Somatic growth rates were calculated from dry body mass as *g* = (ln *M*_t_ − ln *M*_0_) × *t*^−1^, where *M*_t_ is the body mass at the end of the experiment, *M*_0_ is the initial body mass of individuals, and *t* is the time of maturation. The samples in the tin boats were then subjected to elemental analysis.

### Stable isotope analysis

The stable isotope composition of carbon and nitrogen was determined using the Thermo Flash EA1112HT elemental analyzer coupled to a Thermo Delta V Advantage isotope ratio mass spectrometer in the Continuous Flow system. Isotope composition is reported as delta (*δ*) values expressed relative to Vienna-Pee Dee Belemnite (VPDB) for δ^13^C and atmospheric nitrogen for δ^15^N. *δ* values were normalized using a calibration curve based on international standards (USGS 41, USGS 40, and IAEA 600). Measurements were performed with an accuracy of ± 0.12 ‰ for δ^13^ C and ± 0.27 ‰ for δ^15^N, the accuracy assessment was based on repeated measurements of internal standards. Analyses were performed in the Stable Isotope Laboratory of the Institute of Geological Sciences of the Polish Academy of Sciences in Warsaw.

The isotope composition of the algae *Ch. klinobasis* was used as a baseline for calculating isotopic enrichment in experimental animals. Every day during the experiment, a sample (5 mg dry weight of algal particulate organic carbon) from the algal cultures used for feeding animals was analyzed for isotope composition of carbon and nitrogen. Algal suspensions were filtered through the sterile Whatmann GF/C glass fiber filter (Whatmann International Ltd, UK), dried at 60 °C for 48 h, and cooled in a desiccator. Subsamples for isotope analysis were scraped gently from the surface of the filter to the tin boats. Trophic isotope enrichment was calculated using the equation: Δ = *δ*_c_ − *δ*_f_, where *δ*_c_ and *δ*_f_ refer to the carbon and nitrogen isotope compositions of the consumer and food, respectively. Means calculated using daily isotopic composition of algae were used as *δ*_f_.

## Statistical analysis

Factorial ANOVA was used to analyze the effects of fish threat on the life-history parameters of daphnids and on trophic isotope enrichment (Δ). Averages of each parameter for each batch were calculated and used in the analysis as replicates (four replicates per treatment). Life-history data were log-transformed prior to analysis. The model included two factors: Taxon (with two levels, representing each taxon) and Predation (with two levels: fish present, fish absent). Simple regression was used to explore the relationship between predation threat and δ^13^C and δ^15^N values in animals. A significance level of *α* = 0.05 was applied to all statistical analyses. The analyses were performed using Statistica v.13 (Statsoft Inc., USA)—ANOVA, and Statistix v.7 (Analytical Software, USA)—regression analysis.

## Results

**Fig. 2:**
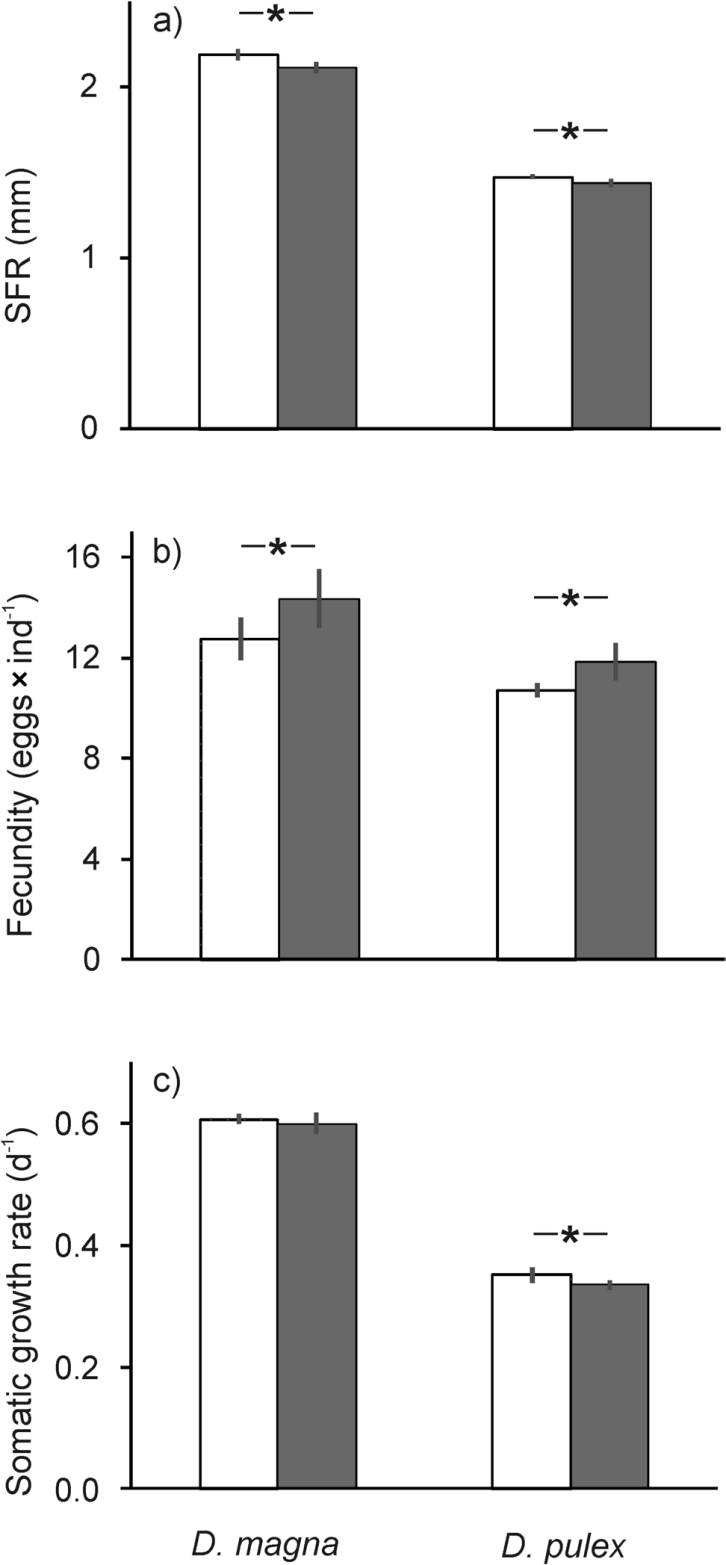
Life history parameters of *Daphnia magna* and *Daphnia pulex* cultivated in the presence (shaded bars) or in the absence (white bars) of a fish threat: **a** size at first reproduction (SFR), **b** fecundity, **c** somatic growth rate. Means from four replicates ± SD. Asterisks indicate statistically significant differences between treatments (post hoc Tukey test, *P* < 0.05)

Individuals of *D. magna* and *D. pulex* exposed to the threat of predation by planktivorous fish reached smaller body sizes (ANOVA *df* = 1, *F* = 26.15, *P* < 0.00007; Tukey test: *p* < 0.0001 for the former taxon and *p* < 0.01 for the latter) and produced more eggs in their first brood (ANOVA *df *= 1, *F* = 11.52, *P* < 0.001; Tukey test: *p* < 0.03 and *P* < 0.0001 respectively), compared to individuals from a predator-free environment (Fig. 2). Species reacted differently to cues of predation (ANOVA *df* = 1, *F* = 6.26, *P* < 0.01): in *D. pulex* somatic growth rates decreased in threatened individuals (Tukey test *p* < 0.0007), whereas in *D. magna* no significant differences were detected in *g* between threatened and control animals (Tukey test *p* = 0.8).

**Fig. 3:**
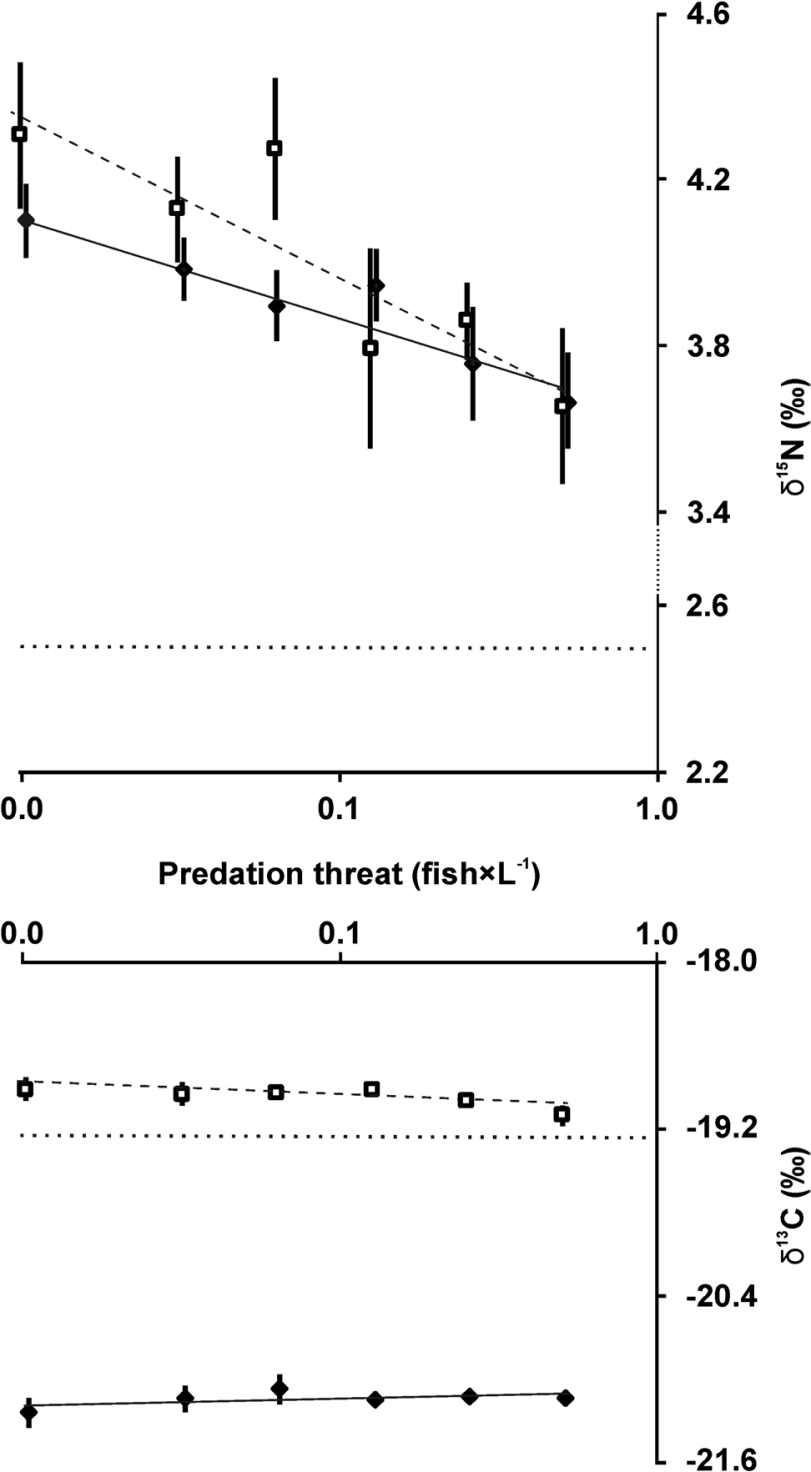
Trophic isotope enrichment of carbon (Δ^13^C) and nitrogen (Δ^15^N) in bodies of *Daphnia magna* (diamonds) and *Daphnia pulex* (rectangles) under fish treat (filled symbols) and in fish-fee control (open symbols). Means from four replicates ± SD. Dotted lines with asterisks indicate statistically significant differences between pairs of treatments (Tukey’s post hoc test, *P* < 0.01)

**Fig. 4:**
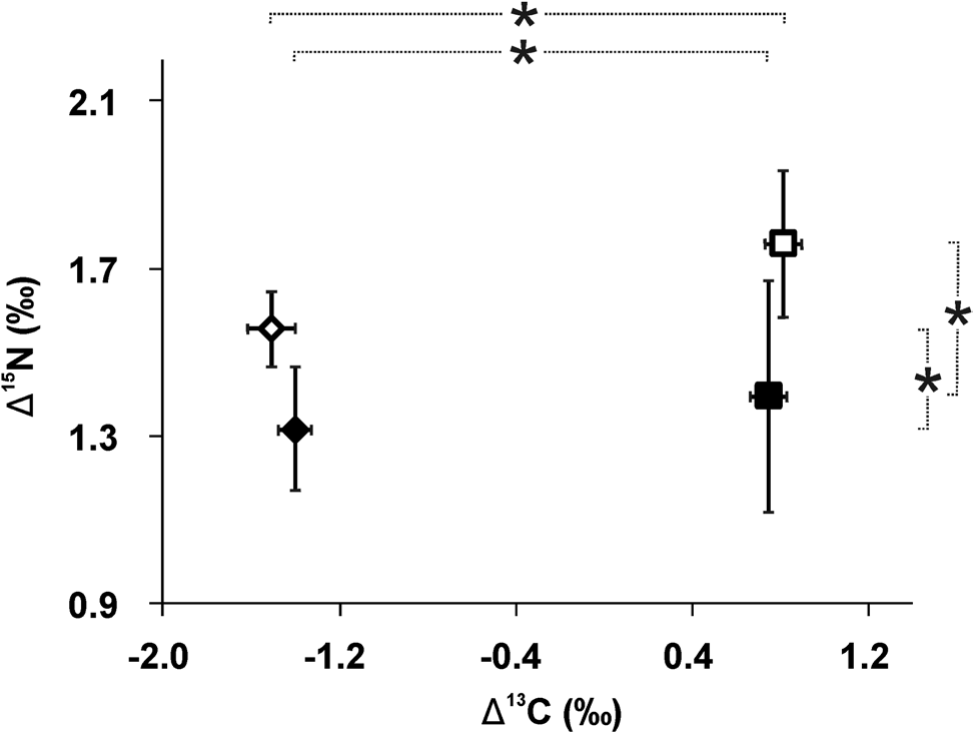
Changes in δ^13^C (upper panel) and δ^15^N (lower panel) of *Daphnia magna* (filled diamonds, solid line) and *Daphnia pulex* (open rectangles, dotted line) in the gradient of predation threat. Means from four replicates ± SD. Horizontal dotted line on each panel represent isotopic ratio of algal food

We did not find evidence that trophic enrichment of carbon isotopes (Δ^13^C) in bodies of daphnids was affected by fish threat (effect Predation Table 1, Fig. 3). Δ^13^C of individuals of *D. magna* and *D. pulex* raised without the signals from foraging predators was not different from Δ^13^C of their fish-threatened conspecifics (post hoc Tukey tests *p* > 0.2 for the former species and *p* > 0.6 for the latter one). However, there were interspecific differences with regard to Δ^13^C between the two tested species (effect Taxon Table 1): *D. pulex*, regardless of whether threatened or not by fish, was higher enriched than *D. magna* (post hoc Tukey test: *p* < 0.0001 and *p* < 0.0001, respectively). δ^13^C of both species of *Daphnia* has not changed with increasing fish threat—the regression lines for both species are almost flat (*D. magna*: δ^13^C = 0.0579 × ln(fish density)—21.16, *R*^2^ = 0.013, *F* = 0.3 *p* > 0.5; *D. pulex*: δ^13^C = − 0.038 × ln(fish density)—18.912, *R*^2^ = 0.529, *F* = 24.7 *p* < 0.0001) (Fig. 4). The regression line for δ^13^C of *D. magna* differed from that of *D. pulex* with regard to slope (*F* = 11.7 *df*_1, 44_ *P* < 0.001) and elevation (*F* = 8189 *df*_1, 45_ *p* < 0.00001).

**Table 1:** Results of ANOVA for the effects of the threat of fish predation on trophic enrichment of carbon isotopes (Δ^13^C) and nitrogen isotopes (Δ^15^N) in *Daphnia*

	*df*	MS	*F*	*P*
Δ^13^C				
Taxon	1	33.4	5035	< 0.00001
Predation	1	0.0036	0.55	0.5
Taxon × predation	1	0.053	8.06	0.006
Error	44	0.29		
Δ^15^N				
Taxon	1	0.13	2.9	0.09
Predation	1	0.60	13.4	< 0.0007
Taxon × predation	1	0.026	0.59	0.4
Error	44	0.045

Trophic enrichment of nitrogen isotopes (Δ^15^N) in bodies of daphnids was affected by fish threat (effect Predation Table 1, Fig. 3). In both species, Δ^15^N of animals subjected to fish threat was lower than that of their non-threatened conspecifics (post hoc Tukey test: *D. magna—p* < 0.002, *D. pulex—p* < 0.01). The two taxa showed no differences between each other with regard to Δ^15^N (effect Taxon, Table 1), regardless of whether threatened or not by fish (post hoc Tukey test *p* > 0.6 and *p* > 0.5, respectively). δ^15^N of both species of *Daphnia* declined with increasing fish threat (*D. magna*: δ^15^N = − 1.216 × ln(fish density) + 4.199, *R*^2^ = 0.523, *F* = 24.2 *p* < 0.0001; *D. pulex*: δ^15^N = − 0.169 × ln(fish density) + 3.5806, *R*^2^ = 0.524, *p* < 0.00006) (Fig. 4). The differences between the regression lines for δ^15^N of *D. magna* and *D. pulex* were statistically insignificant (slopes: *F* = 2.7 *df*_1, 44_ *p* > 0.1; elevations: F = 3.67 df_1, 44_ *p* > 0.06).

## Discussion

It is widely assumed that the isotope composition of a consumer body depends on the isotope composition of food, and it has not been considered that a fear of predation may affect trophic isotope enrichment in consumers. Here, we show for the first time that predation indeed affects isotope ratios and trophic enrichment of nitrogen in consumers. Nitrogen in consumers is generally enriched in the heavier isotope by 3–5‰ (Gannes et al. 1998). In our experiments, δ^15^N values of non-threatened animals (4.1–4.3) fitted to the upper end of the range from the literature reports, while those of fish-threatened (3.5–3.6) fitted to the lower end of the range. With increasing fish threat, δ^15^N of the tested planktonic consumers decreased logarithmically (Fig. 4). Both *Daphnia* species showed similar patterns of response. In animals exposed to predation threat, δ^15^N decreased by 11–15% compared to non-threatened animals, whereas Δ^15^N decreased by 18–21%.

We expected that the isotopic ratio of large-bodied species (*D. magna*) may be more affected than the isotopic ratio of smaller species (*D. pulex*), since large-bodied individuals may be more stressed as they are more conspicuous and more prone to being captured by visually-hunting fish. However, this was not supported by our data, as evidenced by the insignificant interaction between taxon and predation for Δ^15^N (Table 1) and lack of differences between regression lines for δ^15^N.

It is interesting to note that, unlike starvation, which usually affects both δ^13^C and δ^15^N (Boecklen et al. 2011), we have not found evidence that the threat of predation changed δ^13^C of the consumer. It is also very interesting to note that in daphnids threatened by predation, δ^15^N and Δ^15^N decreased. This is contrary to the expectations (we hypothesized that stressed animals would “burn out” their resources and become more depleted in lighter isotope) and contrary to the effects of other stressors (toxins, starvation) which generally increase δ^15^N and Δ^15^N (e.g., Doi et al. 2017; Brzeziński et al. 2020; Helmer et al. 2022). This may indicate that physiological mechanisms different from those in the case of food and toxic distress are responsible for the observed pattern.

Daphnids exposed to the cues of fish predation showed a typical pattern of life-history responses: reduction in body size and increased fecundity (Fig. 2). These reactions are considered adaptive responses, since smaller, less conspicuous individuals are less likely to be spotted by fish, which are visually oriented hunters (Gliwicz 2003). Simultaneously, increased allocation of energy in reproduction allows compensation for increased mortality induced by fish (Gliwicz 2003; Maszczyk and Brzeziński 2018). Daphnids in the experiment were not limited by food supply, and even in the presence of fish cues, they were able to maintain a high somatic growth rate (Fig. 2). Thus, it is not likely that they had to increase catabolic processes—depletion of body reserves is considered the main reason for increased δ^13^C and δ^15^N in starving animals (Boecklen et al. 2011; Shipley and Matich 2020). Differential routing of dietary macromolecules affects isotopic composition of consumers. Depending on whether macromolecules from the diet were incorporated directly into consumer tissue, or used as substrates for synthesis of new macromolecules, isotopic composition of consumer tissues may be more or less similar to that of their diet (Boecklen et al. 2011; Whiteman et al. 2019). Even if dietary macromolecules were directly incorporated, variation in isotope fractionation between a consumer and its diet may occur due to differences in metabolic processing of dietary macromolecules, which depend on environmental context (Whiteman et al. 2019). We suspect that changes in energy allocation from somatic growth to reproduction in response to fish threat may be responsible for the fact that isotope composition was closer to those of algal food, since eggs are rich in storage materials (lipids, proteins). An alternative but non-exclusive explanation may stem from the fact that in daphnids exposed to predation, a reduction of metabolic rates was observed (Rani et al. 2022)—with lower metabolic rates, fractionation of isotopes may be weaker. Further studies, using more advanced methods, e.g., compound-specific analysis of stable isotopes (CSIA, Boecklen et al. 2011; Whiteman et al. 2019), would be necessary to elucidate whether indeed differential allocation of energy acquired from dietary macromolecules was responsible for the observed differences in isotope fractionation between threatened and un-threatened daphnids.

It is also interesting to note that the two species of *Daphnia*, raised in the same conditions and fed the same type and amount of food, developed different δ^13^C values. It was hypothesized that small-bodied, slow-growing animals may show higher isotope enrichment than large-bodied, fast-growing ones (Shipley and Matich 2020). Here, we show that this indeed is the case, at least with regard to δ^13^C values of planktonic cladocerans: *D. magna*, with a larger body size and almost twice as high somatic growth rates as *D. pulex* (Fig. 2) had δ^13^C lower by about 12% and carbon trophic enrichment at least twice lower (Figs. 3, 4). The finding that differences exist in the isotope composition of carbon between zooplankton species that differ with regard to body size has important implications for planning and interpreting field studies of zooplankton relying on the analysis of stable isotopes—body size of animals should be accounted for in such investigations, since using bulk zooplankton, or pooling species of the same genus that differ with regard to body size for the sake of isotope analysis may be a source of bias.

It seems that the predation threat exerts a specific effect on δ^15^N values of pelagic herbivores; at least the outcome is opposite to the effects of stress caused by food limitation (Doi et al. 2017) and pollution (Brzeziński et al. (2020). Moreover, the effect depends on the strength of the threat and seems to be of equal magnitude in at least two species from the genus *Daphnia*. Our findings have three important implications. Firstly, they indicate that δ^15^N values can be potentially used as an indicator of stress caused by predation in natural populations of *Daphnia*. Predation is one of the major ecological drivers of pelagic communities, and representatives of *Daphnia* constitute a keystone guild in freshwater pelagic food webs (Gliwicz 2003; Lampert 2011). Assessment of the predation pressure exerted by fish on this guild in natural conditions is laborious and not easy; it frequently requires additional analysis of fish abundance and fish diet. It may be that δ^15^N values can be used as a proxy for the threat perceived by planktonic prey. This, however, requires further studies examining the effects in the field. The second implication is that the effects of predation on isotope ratios and trophic fractionation of stable isotopes of nitrogen in consumers should be accounted for when using SIA for estimating diet composition or trophic position of consumers. Thirdly, this effect may be applicable to the fossil record for paleoenvironmental reconstruction. Since we showed that δ^15^N changes of < 1‰ in *Daphnia* can be related to the stress exerted by predators, secular δ^15^N variations of < 1‰ in fossil *Daphnia* collected from sediment cores can be used to identify the changes of predation pressure (e.g. introduction or disappearance of planktivorous predators).

## Conclusions

We found that the δ^15^N and Δ^15^N values of daphnids exposed to the threat of predation were different from their non-threatened conspecifics. With increasing perceived predation threat, δ^15^N values decreased logarithmically, and as a consequence, threatened individuals were depleted in ^15^N when compared to their conspecifics not exposed to predation. We found no evidence that δ^13^C and Δ^13^C of daphnids were affected by the predation threat. The two tested species, *D. pulex* and *D. magna*, differed from each other with regard to the carbon isotopes’ composition; nevertheless, in both, the same pattern of response in isotopic composition to the presence of predators was observed. This pattern was different from the effects of other stressors (toxins, starvation) and may specifically reflect the induction of antipredatory defenses in threatened prey. Thus, δ^15^N could possibly be used as an indicator of fish threat to planktonic prey in ecological and paleoecological investigations.

## Supplementary Information

Below is the link to the electronic supplementary material.

Supplementary file1 (PDF 213 KB)

## Data Availability

Data available from the corresponding author on request.
