# Supplementary material for: Predation threat affects isotope ratios of planktonic consumers
Source: Oecologia. 2025 Dec 8;208(1):9. doi: 10.1007/s00442-025-05844-8 (PMC12686068; doi:10.1007/s00442-025-05844-8)
Supplement: Supplementary file 1 — Supplementary file1 (PDF 213 KB) [file 442_2025_5844_MOESM1_ESM.pdf]

**Predation threat affects isotope ratios of planktonic consumers. Characteristics of water used in the experiment.**

Brzeziński, T. [1], Bojanowski M. [2], Radzikowska M.[2]

[1] Department of Hydrobiology, Faculty of Biology, University of Warsaw; Żwirki i Wigury 101, 02-089 Warsaw, Poland

[2] Institute of Geological Sciences, Polish Academy of Sciences; Twarda 51/55, 00-818 Warsaw, Poland

Corresponding author: Tomasz Brzeziński, t.brzezinski@uw.edu.pl, Department of Hydrobiology, University of Warsaw (building CENT3 room 5.04) Żwirki i Wigury 101, 02-089 Warsaw, Poland, phone number +48 22 55 26 518

**Methods**

Samples of control and fish-conditioned medium were analysed for the presence of selected chemicals. Conductivity, concentration of nitrogen in the form of nitrate and ammonium were measured using a YSI EXO-2 multiprameter probe (YSI, USA). Phosphates were measured photometrically according to Koroleff (1983) using a Shimadzu UV/VIS 1200 spectrocolorimeter.

Trace elements in the tested media solutions were measured by inductively coupled plasma mass spectrometry (ICP-MS). Approximately 4 ml of the tested medium was placed in a plastic falcon tube and analysed without dilution in an iCAP RQ ICP-MS (Thermo-Fisher Scientific, Waltham, MA, USA) equipped with a MicroMist borosilicate nebuliser (Glass Expansion, Melbourne, Australia), a Peltier-cooled quartz spray chamber (operating at 3°C), a 2.5 mm ID quartz injector and a removable quartz burner. High sensitivity, high matrix and robust interface skimmer cone inserts were used in this study. Five element mixtures (Certified Reference Material) standards (Sigma-Aldrich, St. Louis, MO, USA) were used to prepare a calibration plot from which limits of detection (LOD) and limits of quantification (LOQ) were determined. Quality control of the ICP-MS system was based on the analysis of appropriate blank samples to verify the purity of the system and on performance tests of the instrument using standard solutions supplied by the manufacturer. In addition, QC samples of known composition and concentration are run during sample analysis to verify that the instrument is operating correctly during the sample sequence.

The analysis of organic compounds content was performed using a gas chromatograph (Thermo Trace 1310) equipped with tandem mass spectrometer TSQ 8000 Evo (Thermo

Fisher Scientific, Bremen, Germany) (GC-MS/MS). Chromeleon 7.26 software was used for data acquisition. Measurements were performed using a TraceGOLD TG-5SilMS 30 m × 0.25 mm × 0.25 µm capillary column (Thermo Fisher Scientific). Analysis was performed using the following temperature program: for HS-SPME-GC-MS analysis the column was heated from 40 to 300°C at a rate of 10°C $min^{-1}$, whereas the maximum temperature was kept for 2 min.

For the analysis of organic extracts the column was heated from 40 to 300°C at a rate of 20°C $min^{-1}$, with the maximum temperature was maintained for 25 min. The injector and transfer line temperatures were 270°C, while the ion source temperature was 250°C. The He carrier gas flow rate was 1 mL $min^{-1}$ . During the analysis, the injector was in splitless mode, and its assigned time of operation was 2 min. The mass spectrometer was operated in electron impact ionization mode at 70 eV. The detector operated at full scan mode with mass range from m/z 40 to 500 and scan time of 180 ms.

Sample preparation for GC-MS/MS analysis involved:

a) HS-SPME-GC/MS: to determine the presence of VOCs (volatile organic compounds) and SVOCs (semi-volatile organic compounds) 2 mL of tested medium were placed into 10 mL vials. The vials were then sealed with aluminum caps containing silicone septum. Then, the samples were placed in a thermostat at 40°C for 10 min, until equilibrium was reached. Afterwards, the sorption process was carried out on the DVB/CAR/PDMS SPME fiber (SUPELCO, Bellefonte, USA). The headspace sorption on the SPME fiber was 15 min at 40°C. HS-SPME-GC/MS analysis did not reveal the presence of any volatile organic compounds.

b) LLE-GC/MS: To determine the presence of non-volatile compounds, 45 mL of tested medium was placed in a glass container, 5 mL of dichloromethane (DCM) was added and

shaken for 15 min. Then the samples were centrifuged (4 kRMP for 5 min) the organic layer was separated, dried under anhydrous magnesium sulfate and analyzed. The analysis of DCM extracts did not reveal the presence of any organic compounds.

c) Concentration - derivatization – GC/MS: To determine the presence of polar compounds 10 mL of tested medium was evaporated to dryness in a gentle stream of nitrogen at 50°C. The residue was then dissolved in 100 µL of acetonitrile (AN) and added 50 µL of BSTFA (derivatization agent). After that sample was heating at 70°C for 1h and analyzed. Analysis of concentrated and derivatizated samples did not reveal the presence of any polar compounds.

The quality control system for GC analyses was based on the analyses of samples of appropriate solvents (blanks) to verify the purity of the chromatographic system and on the analysis of QC samples, which verify both the correctness of the chromatographic system and the MS itself. Such a QC mixture consisted of: trimethylphosphate (CAS 512-56-1) 10 ng/µl, 2,6-dimethylphenol (CAS 576-26-1) 10 ng/µl, 5-Chloro-2-methylaniline (CAS 95-79-4) 10 ng/µl, tri-n-butylphosphate (CAS 126-73-8) 10 ng/µl, dibenzothiophene (CAS 135-65-0) 10 ng/µl, malathion (CAS 121-75-5) 10 ng/µl, methyl stearate (CAS 112-61-8) 10 ng/µl, n-alkanes (C8–C24, even members) 10 ng/µl (all of the compounds of p.a. grade or better, dissolved in dichloromethane).

**Results**

Table 1. Chemical properties of water used in the experiment investigating the effects of predation threat on isotope composition of cladocerans. Abbreviations: R – fish water, C – control (fish-free) water, LOD – limit of detection, LOQ – limit of quantification, BLD – below limit of detection.

| Parameter | | Means | | 95% confidence intervals | | LOD/LOQ | Method |
|---|---|---|---|---|---|---|---|
| | | R | C | R | C | | |
| Conductivity | uS·cm$^{-1}$ | 327 | 332 | 6.9 | 10.8 | | YSI EXO-2 |
| $N-NH_4$ | mg·L$^{-1}$ | 0.041 | 0.039 | 0.003 | 0.003 | | YSI EXO-2 |
| $N-NO_3$ | mg·L$^{-1}$ | 1.11 | 1.19 | 0.2 | 0.05 | | YSI EXO-2 |
| $P-PO_4$ | mg·L$^{-1}$ | 0.0016 | 0.0017 | 0.0002 | 0.0002 | | Koroleff (1983) |
| Organic pollutants | ug·L$^{-1}$ | BLD | BLD | | | LOD: 1-50 ng·L$^{-1}$ | GC-MS/MS |
| Trace elements | | | | | | | |
| Li | ug·L$^{-1}$ | 5.4 | 5.4 | 0.32 | 0.32 | OQ: 66 ng L$^{-1}$ | ICP-MS |
| V | ug·L$^{-1}$ | BLD | BLD | | | LOQ: 7,5 ng L$^{-1}$ | ICP-MS |
| Cr | ug·L$^{-1}$ | BLD | BLD | | | LOQ: 6,3 ng L$^{-1}$ | ICP-MS |
| Co | ug·L$^{-1}$ | 0.04 | 0.04 | 0.0005 | 0.0006 | LOQ: 2,4 ng L$^{-1}$ | ICP-MS |
| Ni | ug·L$^{-1}$ | 0.63 | 0.63 | 0.01 | 0.01 | LOQ: 0,6 ng L$^{-1}$ | ICP-MS |
| Cu | ug·L$^{-1}$ | 4.1 | 4.8 | 0.2 | 0.2 | LOQ: 3,3 ng L$^{-1}$ | ICP-MS |
| As | ug·L$^{-1}$ | BLD | BLD | | | LOQ: 8,7 ng L$^{-1}$ | ICP-MS |
| Se | ug·L$^{-1}$ | BLD | BLD | | | LOQ: 72 ng L$^{-1}$ | ICP-MS |
| Mo | ug·L$^{-1}$ | BLD | BLD | | | LOQ: 1,5 ng L$^{-1}$ | ICP-MS |
| Ru | ug·L$^{-1}$ | BLD | BLD | | | LOQ: 0,3 ng L$^{-1}$ | ICP-MS |
| Rh | ug·L$^{-1}$ | BLD | BLD | | | LOQ: 0,18 ng L$^{-1}$ | ICP-MS |
| Pd | ug·L$^{-1}$ | 0.002 | 0.003 | 5.6E-05 | 2.7E-05 | LOQ: 0,9 ng L$^{-1}$ | ICP-MS |
| Ag | ug·L$^{-1}$ | BLD | BLD | | | LOQ: 2,1 ng L$^{-1}$ | ICP-MS |
| Cd | ug·L$^{-1}$ | BLD | BLD | | | LOQ: 0,3 ng L$^{-1}$ | ICP-MS |
| Sn | ug·L$^{-1}$ | BLD | BLD | | | LOQ: 33 ng L$^{-1}$ | ICP-MS |
| Sb | ug·L$^{-1}$ | 0.02 | 0.02 | 0.0009 | 0.0010 | LOQ: 5,7 ng L$^{-1}$ | ICP-MS |
| Ba | ug·L$^{-1}$ | 20.0 | 18.9 | 0.7 | 0.7 | LOQ: 0,9 ng L$^{-1}$ | ICP-MS |
| Os | ug·L$^{-1}$ | BLD | BLD | | | LOQ: 0,6 ng L$^{-1}$ | ICP-MS |
| Ir | ug·L$^{-1}$ | BLD | BLD | | | LOQ: 0,6 ng L$^{-1}$ | ICP-MS |
| Pt | ug·L$^{-1}$ | BLD | BLD | | | LOQ: 0,6 ng L$^{-1}$ | ICP-MS |
| Au | ug·L$^{-1}$ | BLD | BLD | | | LOQ: 0,9 ng L$^{-1}$ | ICP-MS |
| Hg | ug·L$^{-1}$ | BLD | BLD | | | LOQ: 9,9 ng L$^{-1}$ | ICP-MS |
| Tl | ug·L$^{-1}$ | BLD | BLD | | | LOQ: 0,9 ng L$^{-1}$ | ICP-MS |
| Pb | ug·L$^{-1}$ | BLD | BLD | | | LOQ: 0,6 ng L$^{-1}$ | ICP-MS |

Table 1. continued

| Parameter | | Means | | 95% confidence intervals | | LOD/LOQ | Method |
|---|---|---|---|---|---|---|---|
| | | R | C | R | C | | |
| Trace elements: qualitative analysis | | | | | | | |
| B | ug·L$^{-1}$ | 0.08 | 0.11 | | | LOD: 1 ng·L$^{-1}$ | ICP-MS |
| Sc | ug·L$^{-1}$ | 1.09 | 0.78 | | | LOD: 1 ng·L$^{-1}$ | ICP-MS |
| Ti | ug·L$^{-1}$ | 0.07 | 0.08 | | | LOD: 1 ng·L$^{-1}$ | ICP-MS |
| Zn | ug·L$^{-1}$ | 0.01 | 0.01 | | | LOD: 1 ng·L$^{-1}$ | ICP-MS |
| Ga | ug·L$^{-1}$ | 1.1 | 2.5 | | | LOD: 1 ng·L$^{-1}$ | ICP-MS |
| Ge | ug·L$^{-1}$ | 0.11 | 0.03 | | | LOD: 1 ng·L$^{-1}$ | ICP-MS |
| Br | ug·L$^{-1}$ | 1.4 | 1.9 | | | LOD: 1 ng·L$^{-1}$ | ICP-MS |
| Kr | ug·L$^{-1}$ | 1.2 | 1.2 | | | LOD: 1 ng·L$^{-1}$ | ICP-MS |
| Rb | ug·L$^{-1}$ | 4.3 | 4.5 | | | LOD: 1 ng·L$^{-1}$ | ICP-MS |
| Sr | ug·L$^{-1}$ | 0.13 | 0.15 | | | LOD: 1 ng·L$^{-1}$ | ICP-MS |
| Te | ug·L$^{-1}$ | 0.12 | 0.22 | | | LOD: 1 ng·L$^{-1}$ | ICP-MS |
| I | ug·L$^{-1}$ | 0.48 | 0.44 | | | LOD: 1 ng·L$^{-1}$ | ICP-MS |
| Xe | ug·L$^{-1}$ | 0.18 | 0.20 | | | LOD: 1 ng·L$^{-1}$ | ICP-MS |
| Cs | ug·L$^{-1}$ | 0.01 | 0.05 | | | LOD: 1 ng·L$^{-1}$ | ICP-MS |
| W | ug·L$^{-1}$ | 0.12 | 0.12 | | | LOD: 1 ng·L$^{-1}$ | ICP-MS |
| U | ug·L$^{-1}$ | 0.09 | 0.09 | | | LOD: 1 ng·L$^{-1}$ | ICP-MS |
| Be, Y, Zr, Mn, Nb, In, La, Ce, Pr, Nd, Sm, Eu, Gd, Dy, Ho, Er, Tm, Yb,Lu, Hf, Ta, Re, Bi, Po, At, Rn, Fr, Ra, Ac, Th, Pa, Np, Pu, Am, Cm | ug·L$^{-1}$ | | | | | LOD: 1 ng·L$^{-1}$ | ICP-MS |

**References**

Koroleff F. (1983) Determination of phosphorus. Chemistry of the element in seawater. In: Grashoff K., Ehrhard M., Kremling K. Methods of seawater analysis. Verlag Chemie, Weinham 125-139.
